# Persistent Overactive Cytotoxic Immune Response in a Spanish Cohort of Individuals With Long-COVID: Identification of Diagnostic Biomarkers

**DOI:** 10.3389/fimmu.2022.848886

**Published:** 2022-03-25

**Authors:** Miguel Galán, Lorena Vigón, Daniel Fuertes, María Aránzazu Murciano-Antón, Guiomar Casado-Fernández, Susana Domínguez-Mateos, Elena Mateos, Fernando Ramos-Martín, Vicente Planelles, Montserrat Torres, Sara Rodríguez-Mora, María Rosa López-Huertas, Mayte Coiras

**Affiliations:** ^1^ Immunopathology Unit, National Center of Microbiology, Instituto de Salud Carlos III, Madrid, Spain; ^2^ School of Telecommunications Engineering, Universidad Politécnica de Madrid, Madrid, Spain; ^3^ Family Medicine, Centro de Salud Doctor Pedro Laín Entralgo, Madrid, Spain; ^4^ Biomedical Research Center Network in Infectious Diseases (CIBERINFEC), Madrid, Spain; ^5^ Division of Microbiology and Immunology, University of Utah School of Medicine, Salt Lake City, UT, United States

**Keywords:** Long-COVID, cytotoxic immune response, immune exhaustion, CD8^+^ T cells, NK cells, Random Forest algorithm

## Abstract

Long-COVID is a new emerging syndrome worldwide that is characterized by the persistence of unresolved signs and symptoms of COVID-19 more than 4 weeks after the infection and even after more than 12 weeks. The underlying mechanisms for Long-COVID are still undefined, but a sustained inflammatory response caused by the persistence of SARS-CoV-2 in organ and tissue sanctuaries or resemblance with an autoimmune disease are within the most considered hypotheses. In this study, we analyzed the usefulness of several demographic, clinical, and immunological parameters as diagnostic biomarkers of Long-COVID in one cohort of Spanish individuals who presented signs and symptoms of this syndrome after 49 weeks post-infection, in comparison with individuals who recovered completely in the first 12 weeks after the infection. We determined that individuals with Long-COVID showed significantly increased levels of functional memory cells with high antiviral cytotoxic activity such as CD8^+^ TEMRA cells, CD8^±^TCRγδ^+^ cells, and NK cells with CD56^+^CD57^+^NKG2C^+^ phenotype. The persistence of these long-lasting cytotoxic populations was supported by enhanced levels of CD4^+^ Tregs and the expression of the exhaustion marker PD-1 on the surface of CD3^+^ T lymphocytes. With the use of these immune parameters and significant clinical features such as lethargy, pleuritic chest pain, and dermatological injuries, as well as demographic factors such as female gender and O^+^ blood type, a Random Forest algorithm predicted the assignment of the participants in the Long-COVID group with 100% accuracy. The definition of the most accurate diagnostic biomarkers could be helpful to detect the development of Long-COVID and to improve the clinical management of these patients.

## Introduction

The emergent virus named severe acute respiratory syndrome coronavirus 2 (SARS-CoV-2) is responsible for the coronavirus disease 2019 pandemic (COVID-19), which has caused 281 million infections worldwide and more than 5.4 million deaths to date (1). These numbers are rapidly increasing due to new emergent variants such as Omicron (2).

The clinical outcome of COVID-19 is highly variable, ranging from asymptomatic or mild to a fatal disease. This variability is mostly dependent on how the immune system reacts during the primary infection, due to an exacerbated systemic inflammatory response that has been described in the most severe and critical forms of the disease that may lead to multiple organ dysfunction syndromes (3–5). Those patients who develop severe or critical COVID-19 usually present bilateral pneumonia, respiratory failure with low blood oxygenation, and acute respiratory distress syndrome (ARDS) (6). Other organs may also be compromised, leading to nervous and cardiac system injuries that cause severe complications such as ataxia, acute cerebrovascular disease, olfactory dysfunction, blood hypercoagulability, or cardiomyopathy (7). The median hospital length of stay for patients with severe forms of COVID-19 ranges from less than 1 week to nearly 2 months, whereas the median stay at the intensive care unit (ICU) for those patients with critical COVID-19 varies from 1 to 3 weeks, being shorter for those patients who die from the disease (8). Those hospitalized patients who recover from the most severe forms of the disease may suffer permanent sequelae (9, 10).

The duration of the clinical signs and symptoms may also be very variable among those patients who do not require hospitalization due to asymptomatic or mild COVID-19. In the case of short or acute COVID-19, all signs and symptoms usually disappear from 10 days to 4 weeks after diagnosis (11). However, there is an increasing proportion of patients with mild COVID-19 in which the symptoms do not resolve completely after 4 weeks and may last up to 12 weeks after the clinical onset, then termed Ongoing Symptomatic COVID-19, or even more than 12 weeks, then termed Post-COVID-19 Syndrome (12). These new forms of the disease have been grouped under the term Long-COVID (13, 14), which describes those cases in which the signs and symptoms that continue or develop after acute COVID−19 last from 4 to more than 12 weeks (12). Long-COVID may be developed by 1 in 10 patients who passed COVID-19 (15), and it includes both general and neuropsychiatric persistent symptoms such as cough, fatigue, muscle, and joint pains, insomnia, breathlessness, myalgia, or diarrhea. Other relevant symptoms may compromise the cardiopulmonary system, such as dyspnea, pericarditis, or heart failure (13, 16, 17). Long-COVID may be developed not only by patients who were admitted at the ICU due to critical COVID-19 and now present long-term respiratory sequelae but also by patients with mild COVID-19 who did not require hospitalization.

The causes of Long-COVID are still uncertain, and in most cases, no clear evidence of organ damage is usually found (17). However, some host factors such as gender and age have been related to a higher susceptibility to develop this prolonged syndrome. Women under 50 years of age seem to be more susceptible to present unresolved COVID-19 (18), which indicates a potential role of sex hormones in Long-COVID (19). Another plausible hypothesis could be viral persistence. Although the SARS-CoV-2 viral cycle is different, viral persistence is known to occur in other RNA viruses such as the human immunodeficiency virus (HIV) or hepatitis C virus (HCV), which may produce chronic infection with sustained activation of the immune system (20). Accordingly, prolonged viral shedding has been found in feces from patients with acute COVID-19 after negative PCR in respiratory specimens, suggesting that not only RNA of SARS-CoV-2 but also virions may persist in cells from the gastrointestinal tract (21, 22), likely in enterocytes and small vessels, as was described for the first SARS-CoV (23). The presence of SARS-CoV-2 in the intestinal mucosa could cause intestinal damage that, along with systemic inflammation, may increase bacterial translocation, which would play an essential role in the sustained immune activation and cytokine release syndrome characteristic of COVID-19 (24). On the other hand, several symptoms described in individuals with Long-COVID are quite similar to those developed during autoimmune diseases such as fibromyalgia or chronic fatigue syndrome, which are also linked to persistent inflammation and exacerbated immune responses (25). Individuals with COVID-19 may develop autoantibodies against tissue-associated antigens and immunomodulatory proteins, both soluble such as interferon (IFN) type I or bound to the surface of the immune cells, which would also promote the persistence of the symptoms (26, 27).

In summary, the recovery from COVID-19 seems to be beyond hospital discharge or testing negative for SARS-CoV-2 (28), but the causes for the subsequent perpetuation of the symptoms are still undetermined. To define the clinical guidelines for the prevention, diagnosis, follow-up, and rehabilitation of these individuals, it is necessary to gain a better understanding of the underlying mechanisms of Long-COVID. Our group previously determined that an inefficient cytotoxic response is a reliable biomarker for COVID-19 severity (4). Consequently, in this study, we evaluated the relative importance of this impaired cellular immune response, along with several demographic and clinical features, to develop the persistence of the symptoms and the evolution to Long-COVID by using a Random Forest algorithm.

## Methods

### Study Subjects

Blood samples from 50 individuals who had passed mild, symptomatic COVID-19 during the first pandemic peak in Madrid (Spain) (March–April 2020) were collected in the Primary Healthcare Center Doctor Pedro Laín Entralgo (Madrid, Spain). The inclusion criteria were as follows: over 18 years old, have a positive RT-qPCR assay for SARS-CoV-2 in nasopharyngeal smear or positive titers of virus-specific IgG, and did not require hospitalization while with COVID-19. The subjects were subsequently classified into Long-COVID (n = 30) and Recovered (n = 20). The Long-COVID group included those individuals who had passed mild COVID-19 and referred the presence of at least 8 clinical signs and symptoms compatible with Ongoing Symptomatic COVID-19 or Post-COVID-19 Syndrome according to the National Institute for Health and Care Excellence (NICE) guideline (12) for at least 4 to 12 weeks or more than 12 weeks after the clinical diagnosis of COVID-19. Individuals with Long-COVID were recruited in collaboration with the non-profit Spanish Association of Patients with Long-COVID (Long-COVID-ACTS, Madrid, Spain). The Recovered group included those individuals who had passed acute COVID-19, were homebound until medical discharge, and had the resolution of all signs and symptoms 2 to 4 weeks after the clinical diagnosis. Re-infection or vaccination previous to sampling was not reported by any participant. The most relevant clinical data of all participants with Long-COVID or completely recovered are described in **Supplementary Tables 1, 2**, respectively.

### Ethical Statement

The individuals participating in this study were recruited at the Primary Healthcare Center Doctor Pedro Lain Entralgo (Alcorcón, Madrid, Spain). All participants gave informed written consent to participate, and their anonymity was ensured by the current Spanish and European Data Protection Law. This study was conducted following the Declaration of Helsinki, and it was approved by the Ethical Committee of Instituto de Salud Carlos III (IRB IORG0006384) (CEI PI 07_2021) and the Central Research Commission from the Health Counseling (Comunidad de Madrid, Spain) (favorable report 20210008).

### Cells

Peripheral blood lymphocytes (peripheral blood mononuclear cells (PBMCs)) and plasma were isolated from blood samples by centrifugation through Ficoll–Hypaque gradient (Pharmacia Corporation, North Peapack, NJ, USA) and cryopreserved until the moment of analysis. Due to the low quantity of samples, not all the parameters could be determined in all participants. K562 cell line (ECACC 89121407) was kindly provided by Dr Cristina Eguizabal (Basque Centre Transfusions and Human Tissue, Álava, Spain), and Vero E6 (African green monkey kidney) cell line (ECACC 85020206) was kindly provided by Dr. Antonio Alcami (CBM Severo Ochoa, Madrid).

### Antibodies and Flow Cytometry

Conjugated antibodies CD3-APC, CD4-PercP, CD8-APC-H7, CD8-PercP-Cy5.5, CD16-PercP-Cy5.5, CD25-PE-Cy5, CD56-FITC, CD57-PE, CD107a-PE-Cy7, CD127-FITC, CD158f-BV421, NKG2D-PECy7, NKp44-BUV395, and NKp46-BV650 were purchased from BD Biosciences (San Jose, CA, USA). PD-1-BV650, NKG2A-PE, and NKG2C-AlexaFluor700 were obtained from R&D Systems (Minneapolis, MN, USA). TCRγδ-PE was obtained from BioLegend (San Diego, CA, USA). CD4^+^ and CD8^+^ T-cell memory subpopulations were quantified after staining with CCR7-FITC and CD45RA-PE-Cy7 (BD Biosciences) as follows: naïve (CD45RA^+^CCR7^+^), central memory (TCM) (CD45RA^−^CCR7^+^), effector memory (TEM) (CD45RA^−^CCR7^−^), and terminally differentiated effector memory (TEMRA) (CD45RA^+^CCR7^−^) cells. Data acquisition was performed in a BD LSRFortessa X-20 flow cytometer using FACS Diva software (BD Biosciences). Data analysis was performed with FlowJo_V10 software (TreeStar, Ashland, OR, USA).

For intracellular staining of IFNγ, TNFα and granzyme B (GZB) in CD3^+^CD8^+^ T cells, PBMCs were treated for 4 h at 37°C with PepMix™ SARS-CoV-2 (NCAP) (JPT Peptide Technologies, Berlin, Germany), which contains 102 peptides derived from the Nucleoprotein of SARS-CoV-2, to stimulate the cytotoxic activity of CD8^+^ T cells, in the presence of brefeldin A (BD Biosciences). Cells were then stained with anti-CD3-APC and anti-CD8-PercP. After fixation and permeabilization with IntraPrep Permeabilization Reagent (Beckman Coulter, Brea, CA, USA), cells were stained with antibodies against IFNγ-PE, TNFα-PE, and GZB-FITC (BD Biosciences) and then acquired and analyzed in a BD LSRFortessa X-20 flow cytometer (BD Biosciences) using FACS Diva (BD Biosciences) and FlowJo_V10 software (TreeStar).

### RT-qPCR for Detecting SARS-CoV-2 RNA in Blood

Viral RNA was extracted from the blood samples of all participants of the Long-COVID group using the QIAamp MinElute Virus Kit (Qiagen Iberia, Madrid, Spain). The presence of RNA of SARS-CoV-2 was determined in the blood of all participants in this study following the protocol previously described (29), which is part of the Interim Guidance of the WHO for the diagnostic testing of SARS-CoV-2 (30).

### Pseudotyped SARS-CoV-2 Infection Assay

One-cycle pseudotyped virus encoding SARS-CoV-2 S glycoprotein and the reporter gene *renilla* within an HIV-1 Δ*env* genome (pNL4-3Δ*env*Ren) (31) was used to perform the infection assay in Vero E6 cells. As previously described, cDNA encoding G614 SARS-CoV-2 S glycoprotein (QHU36824.1) without the last 19 amino acids (32) was cloned into pcDNA3.1 expression vector (4). A mutant clone introducing D614G change was generated by site-directed mutagenesis due to D614 viruses being the majority of the earliest variants detected in Spain within clade 19B (33). A monolayer of Vero E6 was infected with identical amounts of both D614 and G614 pseudoviruses (100 ng p24/Gag per p48 well). After 48 h of incubation, Vero cells were cocultured for 1 h with PBMCs isolated from the blood of individuals from our cohort (1:2). Caspase-3 activity was measured in the monolayer as an indicator of cellular cytotoxicity by using the Caspase-Glo 3/7 Assay system (Promega, Madison, WI, USA).

### NK Cell Cytotoxicity Assay

K562 cells that do not express the Major Histocompatibility Complex (MHC) class I molecules on the cell surface (missing self) were used as classical targets of NK cells to evaluate their cytotoxic activity as previously described (4). Briefly, K562 cells were stained with PKH Red Fluorescence Cell Linker kit (Sigma-Aldrich Merck, Darmstadt, Germany) and then cocultured with PBMCs (1:2). After 1 h, they were collected and stained with Annexin V conjugated with fluorescein isothiocyanate (FITC) (Thermo Fisher, Waltham, MA, USA) to quantify early apoptosis by measuring the expression of phosphatidylserine on the cell surface by flow cytometry. BD LSRFortessa X-20 flow cytometer and FACS Diva software were used for data acquisition, and FlowJo_V10 software was used for data analysis.

### Random Forest Algorithm

A Random Forest algorithm (34) was applied to evaluate the accuracy of those demographic, clinical, and immunological parameters that showed significant differences (p < 0.05) in the comparison with the Recovered group to predict the predisposition to develop Long-COVID. The selected parameters were as follows: demographic factors: female gender and O^+^ blood type; clinical factors: lethargy, pleuritic chest pain, dermatological injuries, mean body temperature, dyspnea, diarrhea, conjunctivitis, previous autoimmune diseases, and treatments during COVID-19 such as corticosteroids, antibiotics, and/or vitamin D; immune response factors: total NK cells (CD56^+^), CD3^−^CD56^+^CD16^+^, CD56^+^NKG2A^−^NKG2C^+^, and CD56^+^CD57^+^NKG2C^+^ subpopulations; CD3^+^PD-1^+^; total CD8^+^ T cells, CD8^+^ TEMRA, and CD8^±^TCRγδ^+^ subpopulations; CD4^+^ Tregs; and cytotoxic activity against NK target cells K562 and/or SARS-CoV-2 infected Vero E6 cells. A nested 5-fold cross-validation procedure for each competing algorithm was performed in order to avoid bias in the selection of training, testing, and validation sets, as previously described (35, 36). The relative importance for each feature in the categorization of patients was calculated by using the Gini Variable Importance Measure (VIM) method (37).

### Statistical Analysis

Statistical analysis was performed using Graph Pad Prism 8.0 (Graph Pad Software Inc., San Diego, CA, USA). Quantitative variables were represented as the mean and standard error of the mean (SEM), and significance was analyzed using the unpaired, non-parametric Mann–Whitney test to compare between both Long-COVID and Recovered groups. Qualitative variables were represented as percentages, and significance was analyzed using Fisher’s exact test. p-Values (p) <0.05 were considered statistically significant in all comparisons.

## Results

### Cohorts of Participants

Fifty individuals who passed natural infection by SARS-CoV-2 previous to vaccination were recruited for this study. They were assigned to the Long-COVID group if they showed persistent clinical signs and symptoms from 4 to more than 12 weeks after diagnosis (n = 30), or to the Recovered group if they had resolved all signs and symptoms of COVID-19 in the first 4 weeks after diagnosis. The main demographic and clinical characteristics of all participants are summarized in **Tables 1**, **2** and detailed in **Supplementary Tables 1, 2**. Most individuals in the Long-COVID group (86.6%) were female, whereas 55% of the participants in the Recovered group were female (p = 0.0205). Median age at SARS-CoV-2 acute infection in the Long-COVID and Recovered groups was 42 (interquartile range (IQR) 37–46) and 45 years (IQR 28–57), respectively. Median time from the clinical onset to sampling was 49.7 weeks or 348 days (IQR 150–369 days) in the Long-COVID group versus 11.8 weeks or 83 days (IQR 73–99 days) in the Recovered group. Median length with signs and symptoms compatible with Long-COVID at sampling was 49.7 weeks or 348 days (IQR 150–369 days) and 1.8 weeks or 13 days (IQR 0–49 days), respectively. The most frequent Rh blood group was A^+^ in both cohorts, whereas O^+^ was present in 36.6% of the individuals from the Long-COVID-19 group and 5% from the Recovered group (p = 0.0160). During COVID-19, individuals from the Long-COVID group reported 38.2°C (± 0.66) of recurrent peaks of fever, in comparison with 37.7°C (± 0.18) of peak fever during the first 10 days post-infection in participants from the Recovered group (p = 0.0156). Dyspnea was developed by the majority of participants from the Long-COVID group (86.6%) and 35% of individuals from the Recovered group (p = 0.0002), whereas pleuritic chest pain was present in 76.6% and 25% of the individuals, respectively (p = 0.0005). Additional signs and symptoms with statistical significance reported by individuals from the Long-COVID group, in comparison with individuals from the Recovered group, were lethargy (80%, p < 0.0001), diarrhea (73.3%, p = 0.0037), dermatological injuries (63.3%, p = 0.0003), and conjunctivitis (30%, p = 0.0073). Most participants from the Recovered group did not receive any drug for the treatment of COVID-19, whereas individuals from the Long-COVID group mostly received antibiotics (70%, p = 0.0012), corticosteroids (50%, p < 0.0001), and/or cholecalciferol/vitamin D (40%, p < 0.0001). The most prevalent comorbidities in the group of Long-COVID were autoimmune diseases (30%, p = 0.0366), including psoriasis, rheumatoid arthritis, Hashimoto’s thyroiditis, mixed connective tissue disease (MCTD), and Raynaud’s disease, versus one participant from the Recovered group (5%) who reported both Sjögren’s syndrome and antiphospholipid syndrome prior to SARS-CoV-2 infection.

**Table 1:** Demographic and clinical data of all participants from the Long-COVID group and the Recovered group that were recruited for this study.

	All participants (n = 50)	Long-COVID (n = 30)	Recovered (n = 20)	p-Value
Age (median years, IQR)		42 (37–46)	45 (28–57)	0.9427
Gender: Male		4 (13.4%)	9 (45%)	**0.0205**
Female		26 (86.6%)	11 (55%)	**0.0205**
Time from clinical onset to sampling (median days, IQR)		348 (150–369)	83 (73–99)	**<0.0001**
Time with symptoms (median days, IQR)		348 (150–369)	13 (0–49)	**<0.0001**
Blood group and Rh factor	A^+^	12 (40%)	7 (35%)	0.7737
	A^−^	0 (0%)	2 (10%)	0.1551
	B^+^	1 (3.3%)	1 (5%)	1.0000
	B^−^	0 (0%)	1 (5%)	0.4000
	AB^+^	1 (3.3%)	1 (5%)	1.0000
	AB^−^	0 (0%)	1 (5%)	0.4000
	O^−^	1 (3.3%)	3 (15%)	0.2885
	O^+^	11 (36.6%)	1 (5%)	**0.0160**
	UN	4 (13.3%)	3 (15%)	1.0000
Signs and symptoms during COVID-19	Peak fever (°C) (mean ± SD)	38.2 ± 0.66	37.7 ± 0.18	**0.0156**
	Cough	19 (63.3%)	10 (50%)	0.3927
	Expectoration	13 (43.3%)	3 (15%)	0.0619
	Hemoptysis	2 (6.6%)	1 (5%)	1.0000
	Odynophagia	22 (73.3%)	11 (55%)	0.2293
	Dyspnea	26 (86.6%)	7 (35%)	**0.0002**
	Pneumonia	3 (10%)	3 (15%)	0.6723
	Pleuritic chest pain	23 (76.6%)	5 (25%)	**0.0005**
	Conjunctivitis	9 (30%)	0 (0%)	**0.0073**
	Diarrhea	22 (73.3%)	6 (30%)	**0.0037**
	Malaise	27 (90%)	18 (90%)	1.0000
	Lethargy	24 (80%)	1 (5%)	**<0.0001**
	Migraine	10 (33.3%)	4 (20%)	0.3533
	Arthralgia	23 (76,6%)	11 (55%)	0.1314
	Myalgia	27 (90%)	15 (75%)	0.2400
	Asthenia	29 (96.6%)	18 (90%)	0.5561
	Anosmia	14 (46.6%)	10 (50%)	1.0000
	Ageusia	15 (50%)	10 (50%)	1.0000
	Dermatological injuries	19 (63.3%)	2 (10%)	**0.0003**
Treatment for COVID-19	Hydroxychloroquine	8 (26.7%)	3 (15%)	0.4895
	Antiretroviral drugs	2 (6.7%)	0 (0%)	0.5102
	Corticosteroids	15 (50%)	0 (0%)	**<0.0001**
	Anticoagulants	6 (20%)	1 (5%)	0.2192
	Vitamin D	12 (40%)	0 (0%)	**<0.0001**
	Antibiotics	21 (70%)	4 (20%)	**0.0012**
Comorbidities (risk factors)	Diabetes mellitus	0 (0%)	2 (10%)	0.1551
	Dyslipidemia	9 (30%)	4 (20%)	0.5219
	Arterial hypertension	3 (10%)	1 (5%)	0.6411
	Asthma or COPD	5 (16.6%)	2 (10%)	0.6872
	Cardiovascular disease	1 (3.3%)	2 (10%)	0.5561
	Hypothyroidism	7 (23.3%)	2 (10%)	0.2847
	Autoimmune disease	9 (30%)	1 (5%)	**0.0366**

COPD, chronic obstructive pulmonary disease; UN, unknown; IQR, interquartile range. p-values with statistical significance (p<0.05) are in bold letters.

**Table 2:** Clinical signs and symptoms reported by the participants from the Long-COVID group recruited for this study.

Signs and symptoms	Long-COVID (n = 30)
Dysphagia	12 (40%)
Abdominal pain	15 (50%)
Pyrosis/reflux	19 (63.3%)
Neck/back muscle ache	23 (76.6%)
Headache	26 (86.6%)
Poor concentration	28 (93.3%)
Memory failure	27 (90%)
Bradypsychia	24 (80%)
Cacosmia	11 (36.6%)
Paresthesia	22 (73.3%)
Xerostomia	15 (50%)
Tinnitus	12 (40%)
Dysphonia/aphoniia	17 (56.6%)
Earache	12 (40%)
Hearing loss	6 (20%)
Diplopia	2 (6.6%)
Eye pain	17 (56.6%)
Palpitations	25 (83.3%)
Myocarditis/pericarditis	3 (10%)
Arrhythmia	9 (10%)
T3/T4 levels altered after COVID-19	5 (16.6%)
Diabetes mellitus onset after COVID-19	3 (10%)
Depression	19 (63.3%)
Anxiety	15 (50%)
Insomnia	19 (63.3%)
Hypercoagulability	7 (23.3%)
Alopecia	19 (63.3%)
Nail changes	7 (23.3%)
Petechiae	19 (63.3%)
Urine infection	5 (16.6%)

### Increased Levels of Tregs in the Long-COVID Group

**Figure 1:**
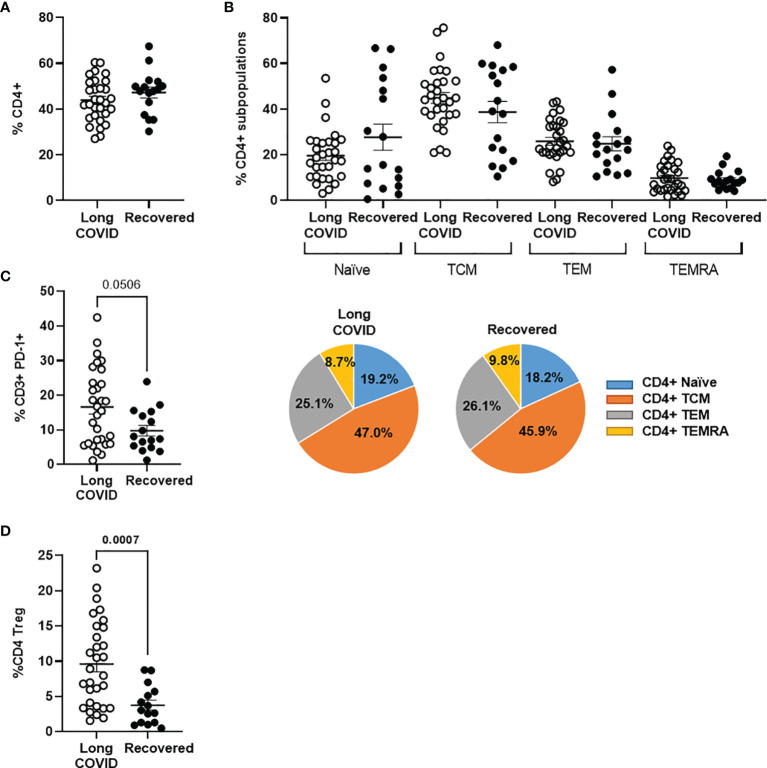
Analysis of CD3^+^ and CD4^+^ T-cell populations in PBMCs from the Long-COVID and Recovered groups. Total levels of CD4^+^ T cells **(A)** and CD4^+^ T-cell memory subpopulations **(B)** were analyzed by flow cytometry. Individual data are shown in a dot plot, and mean data are shown in the pie charts. **(C)** The expression of the exhaustion marker PD-1 was analyzed in CD3^+^ T cells. **(D)** The levels of CD4^+^ Tregs were also quantified by flow cytometry in both groups of individuals. Each dot in the graphs corresponds to one sample, and lines represent mean ± standard error of the mean (SEM). Statistical significance was calculated using non-parametric Mann–Whitney test. PBMCs, peripheral blood mononuclear cells.

We did not find differences in the levels of total peripheral CD4^+^ T cells (**Figure 1A**) or the composition of CD4^+^ memory subpopulations between both Long-COVID and Recovered groups (**Figure 1B**). The total level of lymphocytes (CD3^+^) expressing the immune exhaustion marker PD-1 on the cell surface was increased 1.5-fold in the Long-COVID group, and this difference nearly reached statistical significance (p = 0.0506) (**Figure 1C**). The level of regulatory T cells (Tregs) (CD4^+^CD25^+^CD127^low^) was significantly increased 2.5-fold in the individuals from the Long-COVID group (p = 0.0007), in comparison with the Recovered group (**Figure 1D**). The flow cytometry gating strategy is shown in **Supplementary Figure 1**.

### Changes in CD8^+^ T-Cell Subpopulations in the Long-COVID Group

**Figure 2:**
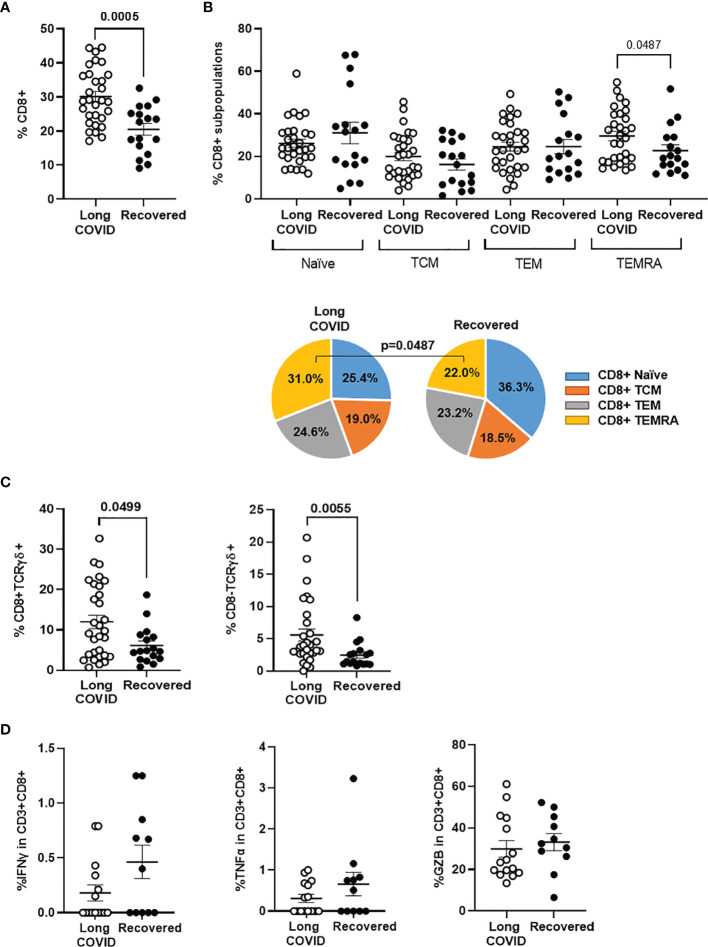
Analysis of CD8^+^ T-cell populations in PBMCs from the Long-COVID and Recovered groups. Total levels of CD8^+^ T cells **(A)** and CD8^+^ T-cell memory subpopulations **(B)** were analyzed by flow cytometry. Individual data are shown in a dot plot, and mean data are shown in the pie charts. **(C)** The levels of CD8 ± TCRγδ^+^ were also determined by flow cytometry in both groups of individuals. **(D)** Quantification of the release of pro-inflammatory cytokines IFNg and TNFa, as well as the serine protease GZB from PBMCs from individuals with Long COVID and the Recovered participants after stimulation with a pool of nucleoprotein peptides from SARS-CoV-2. Each dot in the graphs corresponds to one sample, and lines represent mean ± SEM. Statistical significance was calculated using non-parametric Mann–Whitney test. PBMCs, peripheral blood mononuclear cells.

Total levels of CD8^+^ T cells were increased 1.5-fold in the Long-COVID group, in comparison with the Recovered group (p = 0.0005) (**Figure 2A**). We also observed changes in the distribution of CD8^+^ T-cell memory subpopulations, as the effector CD8^+^ TEMRA cells were increased 1.4-fold in the Long-COVID group (p = 0.00487), whereas the naïve CD8 T cells were reduced 1.4-fold, in comparison with the Recovered group (**Figure 2B**). Regarding the highly cytotoxic CD3^+^ T cells with TCRγδ, both CD8^+^TCRγδ^+^ and CD8^−^TCRγδ^+^ populations were increased 2.0-fold (p = 0.049) and 2.2-fold (p = 0.005), respectively, in the Long-COVID group in comparison with the Recovered group (**Figure 2C**). Stimulation of PBMCs with a pool of nucleoprotein peptides from SARS-CoV-2 showed no significant differences in the capacity of CD8^+^ T cells to release pro-inflammatory cytokines such as IFNγ and TNFα, as well as the serine protease GZB, between individuals with Long-COVID and the Recovered participants (**Figure 2D**). The flow cytometry gating strategy is shown in **Supplementary Figure 2**.

### Increased Levels of NK Cells in the Long-COVID Group

**Figure 3:**
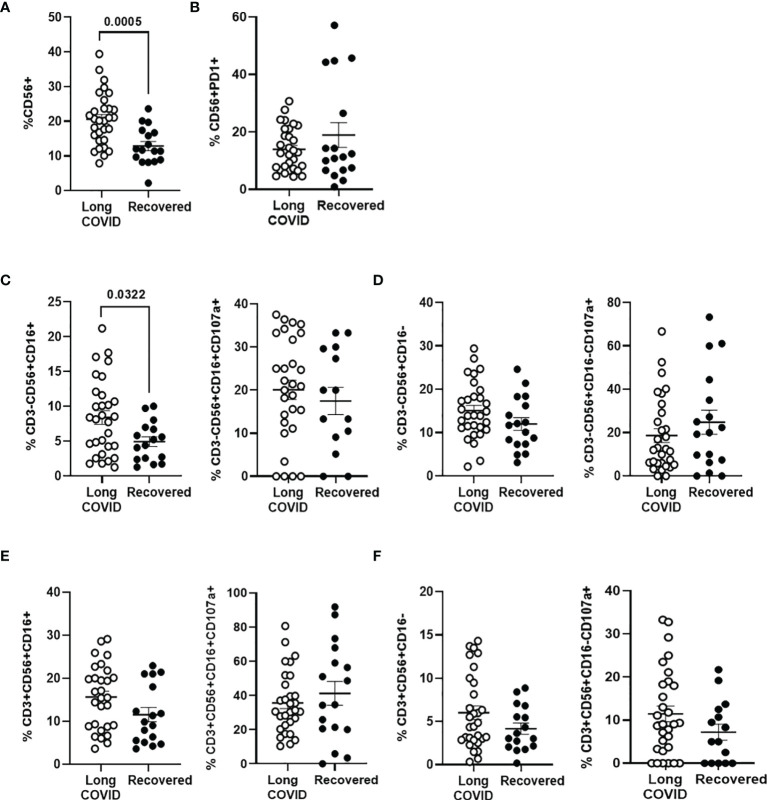
Analysis of NK and NKT cell subpopulations in PBMCs from the Long-COVID and Recovered groups. Total levels of CD56^+^ NK/NKT cells were analyzed by flow cytometry **(A)**, as well as the expression of the exhaustion marker PD-1 in CD56^+^ T cells **(B)**. Total levels of NK cell subpopulation CD56^+^CD16^+^ over CD3^−^ population or with the degranulation marker CD107a over this population CD3^−^CD56^+^CD16^+^ **(C)** and total levels of NK cell subpopulation CD56^+^CD16^−^ over CD3^−^ population or with the degranulation marker CD107a over this population CD3^−^CD56^+^CD16^−^ **(D)** were also analyzed by flow cytometry. Analysis by flow cytometry of the total levels of NKT cell subpopulation CD56^+^CD16^+^ over CD3^+^ population or with the degranulation marker CD107a over this population CD3^+^CD56^+^CD16^+^ **(E)** and total levels of NKT cell subpopulation CD56^+^CD16^−^ over CD3^+^ population or with the degranulation marker CD107a over this population CD3^+^CD56^+^CD16^−^ **(F)**. Each dot in the graphs corresponds to one sample, and lines represent mean ± SEM. Statistical significance was calculated using non-parametric Mann–Whitney test. PBMCs, peripheral blood mononuclear cells.

The total levels of NK cells, characterized by the expression of the activation marker CD56, were increased 1.7-fold (p = 0.0005) in the Long-COVID group, in comparison with the Recovered group (**Figure 3A**). No significant differences were found in the expression of the immune exhaustion marker PD-1 in NK cells between both groups (**Figure 3B**). The population of NK cells expressing CD16 marker on the surface (CD3^−^CD56^+^CD16^+^) was increased 1.7-fold (p = 0.032) in the Long-COVID group (**Figure 3C**, left graph), but no significant differences were found regarding the expression of the degranulation marker CD107a^+^ in this population (**Figure 3C**, right graph). There were no statistically significant differences either between both groups in NK cells not expressing CD16 marker (**Figure 3D**) or in NKT cell populations (**Figures 3E, F**). The flow cytometry gating strategy is shown in **Supplementary Figure 3**.

### Activation Markers of NK Cells in the Long-COVID Group

**Figure 4:**
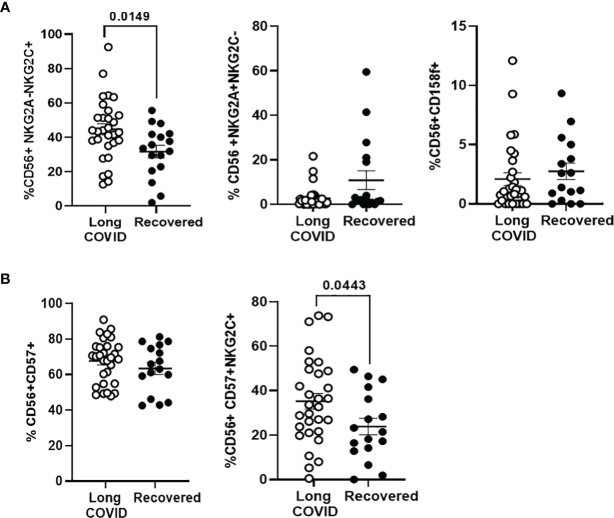
Analysis of the expression of NK cell markers in PBMCs from the Long-COVID and Recovered groups. **(A)** Analysis by flow cytometry of the expression of the activating receptor NKG2C and the inhibitory receptors NKG2A and KIR2DL5/CD158f on the surface of total CD56^+^ cells. **(B)** Analysis by flow cytometry of the expression of the activating receptor NKG2C and the memory marker CD57 on the surface of total CD56^+^ cells. Each dot in the graphs corresponds to one sample, and lines represent mean ± SEM. Statistical significance was calculated using non-parametric Mann–Whitney test. PBMCs, peripheral blood mononuclear cells.

NK cell subpopulation expressing the activation marker NKG2C (CD56^+^NKG2A^−^NKG2C^+^) was increased 1.4-fold (p = 0.0149) in the Long-COVID group, in comparison with the Recovered group (**Figure 4A**, left graph), whereas no significant differences were found between both groups in the NK subpopulation with the inhibitory markers NKG2A (CD56^+^NKG2A^+^NKG2C^−^) (**Figure 4A**, middle graph) or KIR2DL5/CD158f (**Figure 4A**, right graph). There were no differences in the total levels of NK cells with the memory marker CD57 between both groups (**Figure 4B**, left graph), but the population of activated memory NK cells (CD56^+^CD57^+^NKG2C^+^) was increased 1.5-fold (p = 0.044) in the Long-COVID group, in comparison with the Recovered group (**Figure 4B**, right graph). The flow cytometry gating strategy is shown in **Supplementary Figure 4**.

### Persistent Cytotoxic Activity in Peripheral Blood Mononuclear Cells From Individuals of the Long-COVID Group

**Figure 5:**
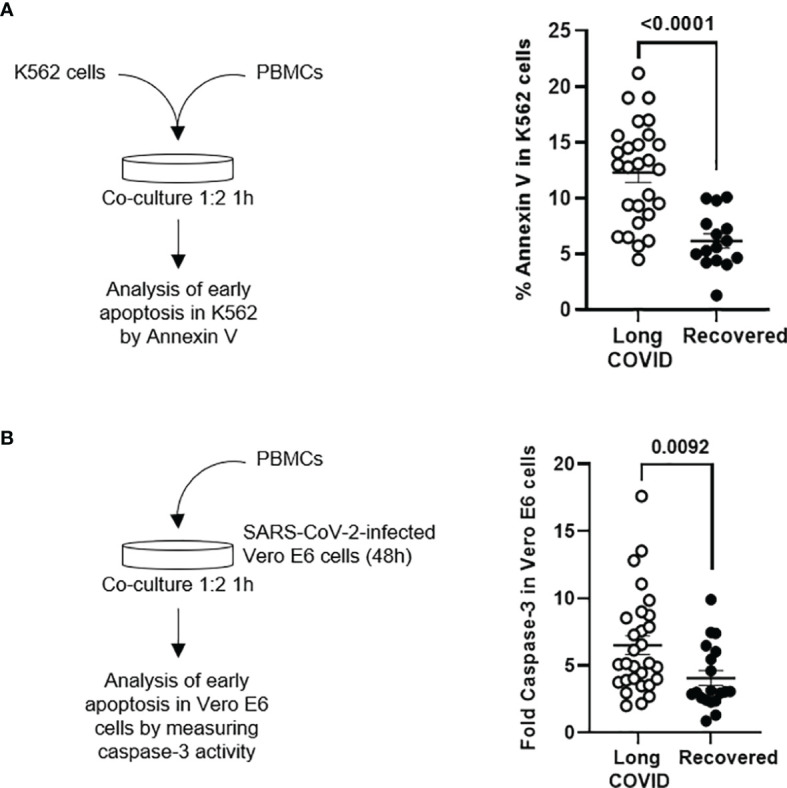
Measurement of cytotoxic activity of PBMCs from the Long-COVID and Recovered groups. **(A)** Diagram (left) and dot plot graph (right) of the assay for the quantification by flow cytometry of Annexin V binding to K562 cells cocultured with PBMCs (1:2) from the Long-COVID and Recovered groups for 1 h **(B)** Diagram (left) and dot plot graph (right) of the assay for the quantification by chemiluminescence of caspase-3 activation in a monolayer of SARS-CoV-2-infected Vero E6 cells cocultured with PBMCs (1:2) from the Long-COVID and Recovered groups for 1 h Each dot in the graphs corresponds to one sample, and lines represent mean ± SEM. Statistical significance was calculated using non-parametric Mann–Whitney test. PBMCs, peripheral blood mononuclear cells.

The cytotoxic activity of PBMCs from the individuals of the Long-COVID group against the classical target of NK cells K562 was increased 2.3-fold (p < 0.0001), in comparison with the Recovered group (**Figure 5A**). The cytotoxic activity of PBMCs was also tested against Vero E6 cells infected with pseudotyped SARS-CoV-2. The activation of caspase-3 was increased 1.7-fold (p = 0.0092) in the monolayer when they were cocultured with PBMCs from individuals of the Long-COVID group, in comparison with the Recovered group (**Figure 5B**).

### Overall Aviremia in Individuals From the Long-COVID Group

All individuals from the Long-COVID group showed an absence of detection of RNA from SAR-CoV-2 in blood, except for one individual (Patient ID 26; see **Supplementary Table 1**) (3.33%) who showed amplification of RNA in blood at cycle threshold (CT) 32.97 (data not shown).

### Application of Random Forest for the Evaluation of Diagnostic Biomarkers for Long-COVID

**Figure 6:**
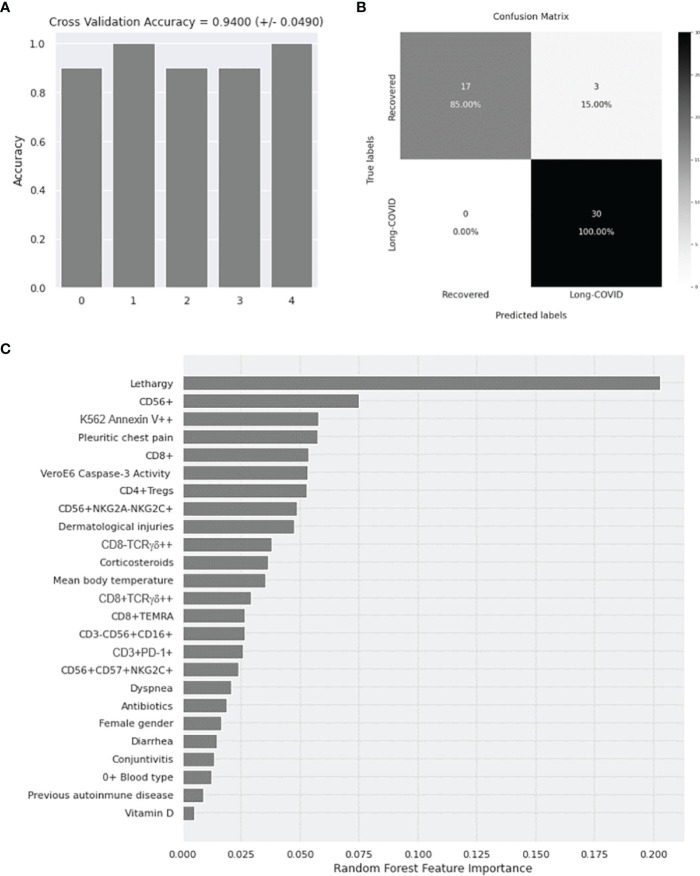
Application of Random Forest algorithm and Gini VIM method for the evaluation of the importance of demographic, clinical, and immunological parameters with statistical significance as diagnostic biomarkers for Long-COVID. **(A)** Calculation of the accuracy for 5 iterations of the outer loop of the nested K-fold cross validation. **(B)** Confusion matrix confronting the conditions predicted by the algorithm and the true conditions for the correct assignment of the participants to the Long-COVID group or the Recovered group. **(C)** Classification of the demographic, clinical, and immunological features with statistical significance according to their importance to predict the correct classification of the individuals to the Long-COVID group or the Recovered group. VIM, Variable Importance Measure.

An accuracy of 94% ± 4.90% was obtained for the 5 iterations of the outer loop of the nested K-fold cross validation for each competing algorithm (**Figure 6A**). As a result, all 30 patients (100%) in the Long-COVID group were correctly assigned to this group, whereas 17 individuals of 20 (85%) were correctly assigned to the Recovered group (**Figure 6B**). The Gini VIM method determined that clinical parameters such as lethargy, pleuritic chest pain, dermatological injuries, treatment with corticosteroids, immune factors (such as enhanced total levels of NK cells, CD8^+^ T lymphocytes, and Tregs), and increased cytotoxic activity were the most important variables to assign the individuals to the Long-COVID group (**Figure 6C**), whereas demographic parameters such as female gender and O^+^ blood type, or previous history of autoimmune disease, were within the variables with less importance.

## Discussion

Long-COVID is emerging as a new relevant syndrome worldwide that is characterized by the maintenance of long-term signs and symptoms of COVID-19 that are not resolved in the first 4 weeks after the infection (12). Several host and viral factors may be involved in the development of Long-COVID, although the underlying basic mechanisms are still unknown. To determine the most important host factors that could be used as diagnostic parameters to evaluate the development of Long-COVID, we collected and analyzed essential demographic, clinical, and immunological characteristics in a Spanish cohort of individuals with signs and symptoms compatible with Long-COVID in comparison with individuals who recovered completely before 4 weeks after the infection. A Random Forest algorithm was applied to identify the most important variables to predict the susceptibility to develop Long-COVID.

Regarding the host factors that might be related to Long-COVID, some demographic characteristics such as age and gender have been appointed as contributing host factors for an increased susceptibility (11, 19, 38). Before the general vaccination against COVID-19, most patients admitted to the ICU due to a critical form of the disease were usually men over 65 years old (39). In contrast, most participants in our cohort of Long-COVID were women under 45 years old, which is in accordance with previous studies (18, 40). Rh blood O^+^ type was also significantly more frequent in the individuals of the Long-COVID group, but we cannot rule out that most Spanish populations present this blood type (41). Although it has been reported that individuals with Rh^−^ blood type may be at lower risk of SARS-CoV-2 infection and development of severe illness or death by COVID-19 (42, 43), other studies did not find a significant impact of Rh on COVID-19 severity (44, 45). In our study, according to the Random Forest analysis, both female gender and Rh blood type were within the less important possible diagnostic factors for Long-COVID. However, clinical signs and symptoms such as persistent lethargy, pleuritic chest pain, and recurrent dermatological injuries showed more relative importance for the prediction of higher susceptibility to Long-COVID.

Considering the potential viral factors that might be involved in the development of Long-COVID, one hypothesis that should be taken into account is that SARS-CoV-2 might find mechanisms to persist in the organism after the acute infection. In this case, Long-COVID syndrome would be a consequence of an unresolved SARS-CoV-2 infection (46). A prolonged SARS-CoV-2 viral shedding in feces has been described several months after the COVID-19 diagnosis (21, 47). This suggests that SARS-CoV-2 may persist in sanctuaries such as the epithelial cells of the gastrointestinal tract or the tissue macrophages in the gut-associated lymphoid tissue (GALT) (48, 49), which would explain not only the continuous viral shedding but also the gastrointestinal symptoms developed by individuals with Long-COVID (50). In fact, alterations in the gut microbiota have been described in individuals with Long-COVID even 6 months after the infection (51). We cannot rule out this possibility in our cohort, as at least 70% of individuals with Long-COVID had been treated with broad-spectrum antibiotics. However, SARS-CoV-2 could also persist in other anatomical sanctuary organs with immune privilege such as the central nervous system (CNS) (52) or in other organs and cells in which ACE2 receptor is highly expressed such as the intestine, kidneys, cardiac tissue, reproductive system, thyroid, gallbladder, and nasal mucosa (53, 54), which would also explicate the highly variable signs and symptoms that are associated with Long-COVID (55). The persistence of SARS-CoV-2 in the organism would also correlate with the recurrent peaks of fever reported by the participants from the Long-COVID group, which was significantly increased at 0.5°C in comparison with the individuals from the Recovered group, in which the fever stopped once the infection was resolved. Although more studies will be necessary to determine whether the virus may be found in the feces of these individuals, low SARS-CoV-2 viremia was detected in one participant from the Long-COVID group (Patient ID 26). This participant was a 44-year-old woman, A^+^, with previous history of asthma and chronic obstructive pulmonary disease (COPD) who was treated with azithromycin during the first days after COVID-19 diagnosis, had signs and symptoms compatible with Long-COVID at the time of sampling (21.4 weeks post-infection), and did not report a subsequent re-infection after the first exposure to SARS-CoV-2. She informed about persistent neurological symptoms and diarrhea, which would support the possibility of a viral persistence in the CNS or in the GALT, with occasional viremia.

The persistence of SARS-CoV-2 or its viral products in the organism would then contribute to the increased antiviral, cytotoxic activity detected in individuals from the Long-COVID group, along with the associated gastrointestinal symptoms, which were likely also due to the presence of activated immune cells in the GALT (56). Persistent epithelial damage due to a chronic inflammatory response has been appointed as a possible cause of Long-COVID (57). It is noteworthy that patients with critical COVID-19 who are admitted to the ICU usually present CD4^+^ cytopenia (4, 58), which makes it difficult to develop a complete, effective immune response against SARS-CoV-2. However, the individuals from the Long-COVID group in our study showed not only normal levels of CD4^+^ T cells in peripheral blood but also CD8^+^ lymphocytosis with an increased proportion of TEMRA cells, which are highly differentiated effector cells essential for CD8^+^ function (59). This enhanced terminal differentiation of memory CD8^+^ T cells pointed at the presence of a potent antiviral immune response that was supported by the presence of increased levels of highly cytotoxic populations of CD8^±^ T lymphocytes expressing TCRγδ. These cells have been related to a potent antiviral and antineoplastic response, and therefore, they are currently the target of different strategies to reprogram their cytotoxic potential against specific targets such as viruses or cancerous cells (60, 61). Although the capacity of CD8^+^ T cells to produce pro-inflammatory cytokines and GZB was not significantly different between individuals with Long-COVID and those who completely recovered, the sustained presence of these cytotoxic cell populations from the adaptive immune system indicated that the participants from the Long-COVID group had developed a potent memory response against SARS-CoV-2 that was still fully active after more than 49 weeks post-infection, whereas this response had waned in the Recovered group once the infection was cleared. The increased expression of exhaustion markers such as PD-1 in CD3^+^ T lymphocytes from these individuals of the Long-COVID group could be a consequence of the continuous activation of the immune system. The PD-1/PD-L1 axis is upregulated during acute viral infection and after infection with persistent RNA viruses such as HIV and HCV, and it has been related to viral evasion of the immune system and prolonged inflammatory responses (62). Moreover, the significant increase of Tregs observed in PBMCs from individuals of the Long-COVID group may indicate the failed attempt of the immune system to control this persistent immune response.

The long-term activation of the immune system in response to triggers such as pathogens, vaccines, drugs, or chemicals has been previously related to the development of autoimmune diseases due to the loss of tolerance (63). The link between the development of autoimmunity and a previous infection is not always clear, as occurs with common autoimmune diseases such as lupus erythematosus, multiple sclerosis, type 1 diabetes mellitus, and rheumatoid arthritis. However, the discovery of autoantibodies in individuals with Long-COVID may link this syndrome not only to a persistent viral infection but also to an immune dysregulation that would lead to a sustained targeting of own cells (26, 64). The detection of high levels of functional cytotoxic memory cells in peripheral blood of the participants from the Long-COVID group may explain why several signs and symptoms of this syndrome resembled those of an autoimmune disease. Some previous predisposition may be then contributing since the most prevalent comorbidities showed by these individuals were autoimmune diseases, although they were not considered within the most accurate diagnostic biomarkers for Long-COVID in our Random Forest model. However, the hypothesis that a persistent memory cytotoxic response against SARS-CoV-2 could be the cause for the development of Long-COVID was supported in our cohort by the presence not only of high levels of CD8^+^ TEMRA cells but also of NK cells expressing both memory (CD57) and activation (NKG2C) markers that did not waste and wane after SARS-CoV-2 infection was cleared, despite the presence of high levels of Tregs. Moreover, these immunological factors were considered the most important variables for the prediction of a higher susceptibility to develop Long-COVID in our Random Forest model.

The difference in the median time from the clinical onset to sampling in the Long-COVID group versus the Recovered group could be considered a potential limitation of our study. However, all participants were infected with SARS-CoV-2 during the same period of time that was the first pandemic peak in Spain (March to April 2020), and whereas all signs and symptoms of COVID-19 were completely resolved at the time of sampling (12 weeks post-infection) in all participants of the Recovered group, the individuals of the Long-COVID group still presented recurrent signs and symptoms of the disease more than 49 weeks post-infection. Therefore, all inclusion criteria were met in both groups.

In conclusion, Long-COVID syndrome could be the consequence of a long-lasting memory cytotoxic immune response that had been triggered by SARS-CoV-2, or its viral products are hidden in some anatomical sanctuaries such as the CNS or tissue macrophages in the GALT, where it would be protected from clearance by the immune system. However, we also have to consider that Long-COVID shows characteristics of autoimmune disease and that infected individuals may be more susceptible to developing this syndrome due to the activation of an exacerbated memory immune response that cannot be adequately controlled by homeostatic mechanisms once the infection was cleared. Having a better understanding of the underlying mechanisms of Long-COVID could be helpful to prevent the development of this syndrome and to improve the clinical management of these patients. According to our Random Forest model, 100% of the participants included in the Long-COVID group were correctly assigned to this group using parameters related to long-lasting cytotoxicity, which proved their validity as biomarkers to diagnose the development of this syndrome.

## Data Availability Statement

The original contributions presented in the study are included in the article/**Supplementary Material**. Further inquiries can be directed to the corresponding authors.

## Ethics Statement

The studies involving human participants were reviewed and approved by the Ethical Committee of Instituto de Salud Carlos III (IRB IORG0006384) (CEI PI 07_2021) and the Central Research Commission from the Health Counseling (Comunidad de Madrid, Spain) (favorable report 20210008). The patients/participants provided their written informed consent to participate in this study.

## Contributing Members of the Multidisciplinary Group of Study of COVID-19 (MGS-COVID)

Esther Alonso Herrador^1^, Pablo Amich Alemany^1^, Victoria Bosch Martos^1^, Lorena Cordova-Castaño^1^, Aurora Expósito Mora^1^, Javier García-Pérez^2,3^, María Mercedes Gea Martinez^1^, Alberto Gomez Bonilla^1^, María Victoria Leon Gomez^1^, Gema Lora Rey^1^, Maria Luisa Muñoz Balsa^1^, Javier Pérez Gonzalez^1^, Sandra Pérez-Santos^1^, Jose Sanchez Hernández^1^, Andrea Vinssac Rayado^1^

^1^Centro de Salud Doctor Pedro Laín Entralgo, Alcorcón, Spain.

^2^AIDS Immunopathology Unit, National Center of Microbiology, Instituto de Salud Carlos III, Majadahonda, Madrid, Spain.

^3^Biomedical Research Center Network in Infectious Diseases (CIBERINFEC), Spain

## Author Contributions

MC and ML-H conceptualized the project. MG, LV, MC, and ML-H wrote the manuscript. LV, SR-M, FR-M, MT, GC, and EM processed and stored all blood samples. MM-A, SD-M, and MT identified, selected, and recruited the patients. MG, LV, GC, MT, EM, and SR-M performed the analytical experiments. LV and MG collected and analyzed the clinical data and laboratory results. DF and VP performed the Random Forest analysis. All co-authors read and approved the final version of the manuscript.

## Funding

This work was supported by the Coordinated Research Activities at the National Center of Microbiology (CNM, Instituto de Salud Carlos III) (COV20_00679) to promote an integrated response against SARS-CoV-2 in Spain (Spanish Ministry of Science and Innovation), which is coordinated by Dr Inmaculada Casas (WHO National Influenza Center of the CNM); a generous donation provided by Chiesi España, S.A.U. (Barcelona, Spain); the Spanish Ministry of Science and Innovation (PID2019-110275RB-I00); and the Spanish AIDS Research Network RD16CIII/0002/0001 that is included in Acción Estratégica en Salud, Plan Nacional de Investigación Científica, Desarrollo e Innovación Tecnológica 2016-2020, Instituto de Salud Carlos III, European Region Development Fund (ERDF). The work of ML-H and SR-M is financed by NIH grant R01AI143567. The work of MT is supported by Instituto de Salud Carlos III (COV20_00679). The work of LV is supported by a pre-doctoral grant from Instituto de Salud Carlos III (FIS PI16CIII/00034-ISCIII-FEDER). The work of FR-M is financed by the Spanish Ministry of Science and Innovation (PID2019-110275RB-I00).

## Conflict of Interest

The authors declare that the research was conducted in the absence of any commercial or financial relationships that could be construed as a potential conflict of interest.

## Publisher’s Note

All claims expressed in this article are solely those of the authors and do not necessarily represent those of their affiliated organizations, or those of the publisher, the editors and the reviewers. Any product that may be evaluated in this article, or claim that may be made by its manufacturer, is not guaranteed or endorsed by the publisher.
