# Supplementary material for: Persistent Overactive Cytotoxic Immune Response in a Spanish Cohort of Individuals With Long-COVID: Identification of Diagnostic Biomarkers
Source: Front Immunol. 2022 Mar 25;13:848886. doi: 10.3389/fimmu.2022.848886 (PMC8990790; doi:10.3389/fimmu.2022.848886)
Supplement: Supplementary file 3 [file Presentation_3.pptx]

## Slide 1
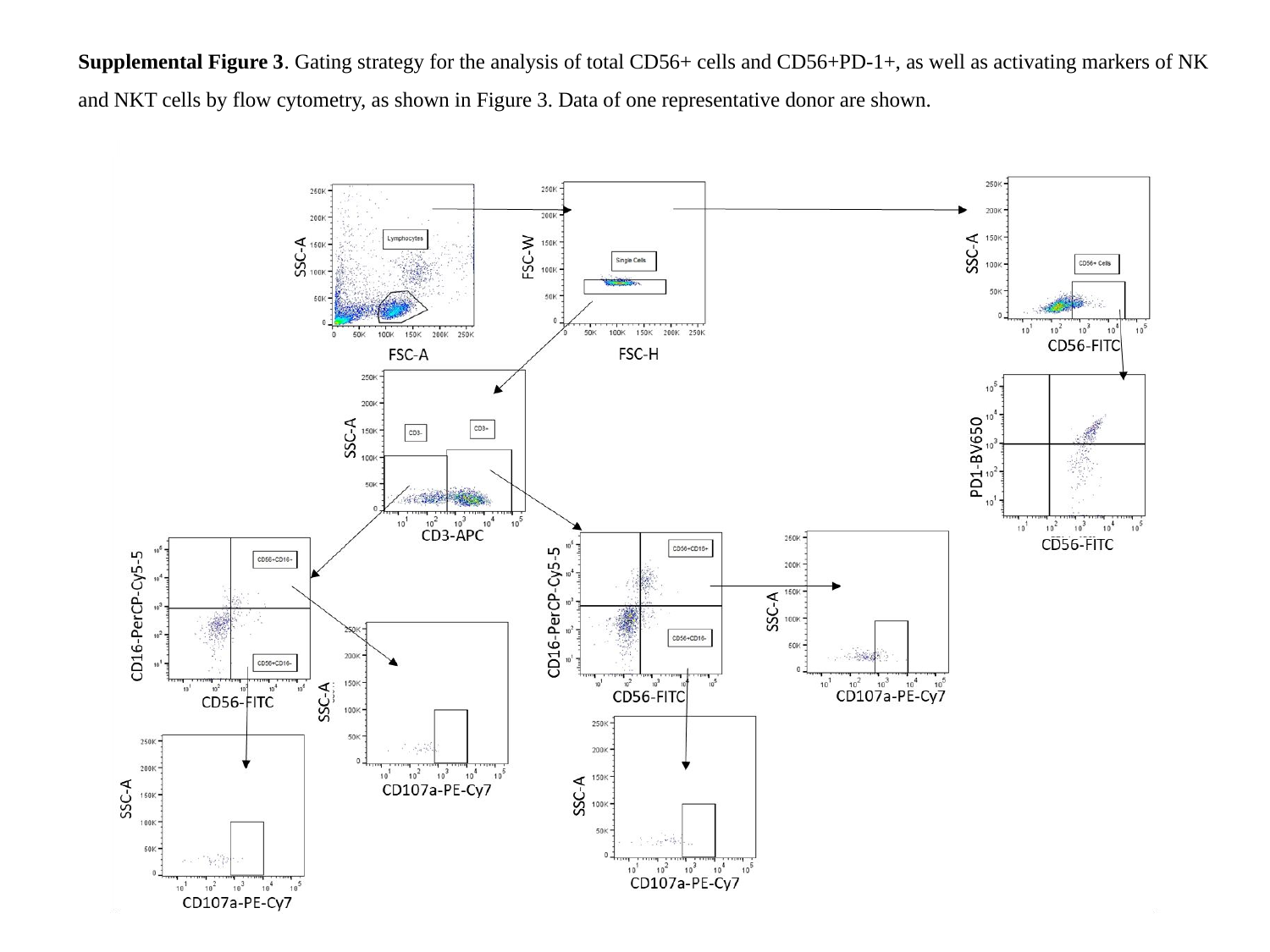

Supplemental Figure 3. Gating strategy for the analysis of total CD56+ cells and CD56+PD-1+, as well as activating markers of NK and NKT cells by flow cytometry, as shown in Figure 3. Data of one representative donor are shown.
