# Supplementary material for: Persistent Overactive Cytotoxic Immune Response in a Spanish Cohort of Individuals With Long-COVID: Identification of Diagnostic Biomarkers
Source: Front Immunol. 2022 Mar 25;13:848886. doi: 10.3389/fimmu.2022.848886 (PMC8990790; doi:10.3389/fimmu.2022.848886)
Supplement: Supplementary file 4 [file Presentation_4.pptx]

## Slide 1
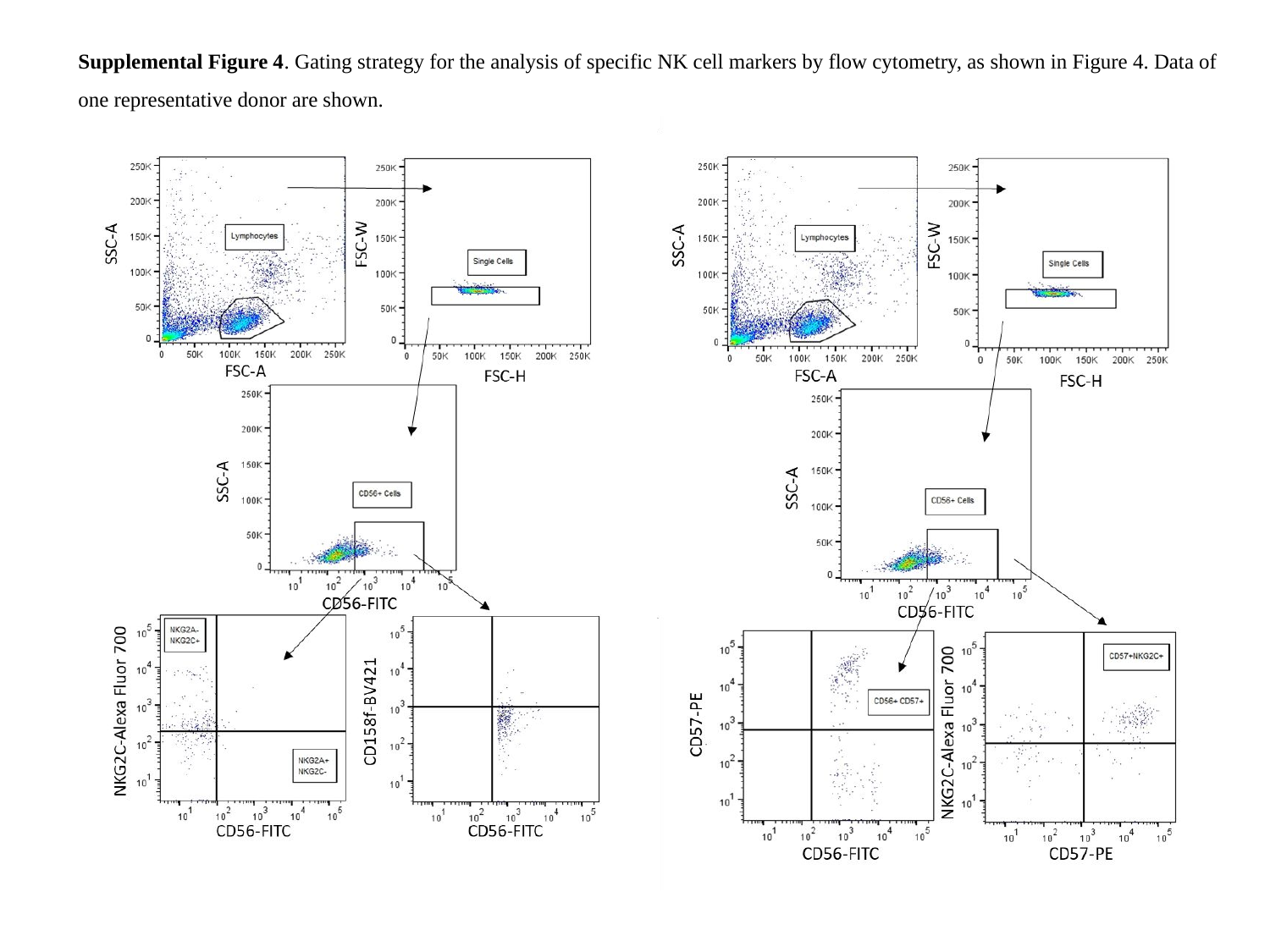

Supplemental Figure 4. Gating strategy for the analysis of specific NK cell markers by flow cytometry, as shown in Figure 4. Data of one representative donor are shown.
