# Supplementary material for: Persistent Overactive Cytotoxic Immune Response in a Spanish Cohort of Individuals With Long-COVID: Identification of Diagnostic Biomarkers
Source: Front Immunol. 2022 Mar 25;13:848886. doi: 10.3389/fimmu.2022.848886 (PMC8990790; doi:10.3389/fimmu.2022.848886)
Supplement: Supplementary file 5 [file Table_1.docx]

**Supplemental Table 1.** Clinical characteristics of non-hospitalized patients with Long COVID-19 and recruited at the Primary Healthcare Center Doctor Pedro Laín Entralgo (Madrid, Spain) with the collaboration of non-profit Spanish Association of Patients with Long-COVID (Long-COVID-ACTS). The duration of signs and symptoms was divided in periods of less than 3 months (12 weeks), between 3 and 6 months, between 6 and 9 months, or more than 9 months.

| **Patient's ID** | **Age (years)** | **Gender**  **(M/F)** | **Blood Group**  **(ABO, Rh±)** | **Days from clinical onset to sample** | **Days with COVID-19 symptoms** | **SARS-CoV-2 qRT-PCR** | **SARS-CoV-2 IgGs** | **Signs and symptoms during acute COVID-19** | | | | | | | | | |
| --- | --- | --- | --- | --- | --- | --- | --- | --- | --- | --- | --- | --- | --- | --- | --- | --- | --- |
|  |  |  |  |  |  |  |  | **Fever**  **(ºC)** | **Oxygenation (%)** | **Cough**  **(months)** | **Expecto**  **ration**  **(months)** | **Hemoptysis**  **(months)** | **Malaise**  **(months)** | **Lethargy**  **(months)** | **Migraine** | **Arthalgia**  **(months)** | **Myalgia**  **(months)** |
| 1 | 33 | F | UN | 346 | 346 | YES | YES | 38 | 88 | NO | NO | NO | >9 | >9 | NO | NO | >9 |
| 2 | 31 | F | B + | 352 | 352 | NO | YES | UN | UN | NO | NO | NO | >9 | >9 | YES | >9 | >9 |
| 3 | 45 | F | A + | 347 | 347 | YES | YES | 38 | 99 | >9 | NO | NO | >9 | <3 | NO | NO | NO |
| 4 | 48 | F | UN | 358 | 358 | YES | YES | 37.8 | 96 | >9 | NO | NO | 6-9 | 3-6 | NO | >9 | >9 |
| 5 | 43 | M | UN | 342 | 342 | YES | YES | UN | UN | NO | NO | NO | >9 | NO | YES | >9 | >9 |
| 6 | 42 | F | A + | 349 | 349 | YES | YES | 38 | 92 | 6-9 | <3 | NO | >9 | 3-6 | YES | >9 | >9 |
| 7 | 55 | F | O- | 348 | 348 | NO | YES | UN | UN | NO | 6-9 | NO | >9 | NO | NO | >9 | >9 |
| 8 | 50 | F | O + | 192 | 192 | YES | NO | 39.7 | UN | 6-9 | 6-9 | NO | 6-9 | NO | NO | 6-9 | 6-9 |
| 9 | 33 | M | O + | 336 | 336 | NO | YES | 38 | 96 | 3-6 | >9 | NO | NO | >9 | YES | NO | <3 |
| 10 | 57 | F | O + | 351 | 351 | YES | YES | 38 | 92 | <3 | <3 | NO | >9 | >9 | NO | >9 | >9 |
| 11 | UN | F | A + | 194 | 194 | YES | NO | 38.5 | 93 | >9 | NO | >9 | >9 | >9 | NO | >9 | >9 |
| 12 | 46 | F | O + | 345 | 345 | YES | YES | UN | UN | NO | NO | NO | 3-6 | <3 | NO | >9 | >9 |
| 13 | 42 | F | O + | 341 | 341 | YES | YES | 37.7 | 93 | NO | NO | NO | >9 | >9 | YES | >9 | >9 |
| 14 | 46 | F | A + | 349 | 349 | YES | NO | 38 | 90 | 3-6 | NO | NO | >9 | >9 | NO | >9 | >9 |
| 15 | 41 | F | A + | 347 | 347 | NO | YES | UN | 88 | >9 | NO | NO | >9 | >9 | NO | <3 | 6-9 |
| 16 | 37 | F | O + | 311 | 311 | YES | YES | 38 | 93 | NO | NO | NO | >9 | NO | YES | >9 | >9 |
| 17 | 42 | F | O + | 350 | 350 | NO | YES | 40 | 90 | NO | NO | NO | >9 | >9 | NO | >9 | >9 |
| 18 | 40 | F | O + | 358 | 358 | YES | YES | 38.5 | 94 | >9 | 6-9 | NO | >9 | >9 | NO | 6-9 | >9 |
| 19 | 33 | M | A+ | 356 | 356 | YES | YES | UN | UN | NO | NO | NO | NO | NO | NO | NO | NO |
| 20 | 33 | F | A + | 171 | 171 | YES | NO | 37.5 | 91 | NO | NO | NO | 3-6 | 3-6 | NO | NO | 3-6 |
| 21 | 39 | F | O + | 348 | 348 | YES | YES | 38.6 | UN | >9 | >9 | NO | >9 | >9 | YES | >9 | >9 |
| 22 | 42 | F | A + | 358 | 358 | YES | YES | 37.7 | 92 | 3-6 | NO | NO | >9 | 3-6 | NO | NO | 6-9 |
| 23 | 44 | F | A + | 369 | 369 | NO | YES | 37.7 | 95 | 3-6 | >9 | NO | >9 | >9 | YES | >9 | >9 |
| 24 | UN | F | A + | 352 | 352 | YES | YES | 37.7 | 94 | 3-6 | <3 | NO | >9 | >9 | YES | >9 | >9 |
| 25 | 52 | F | AB + | 366 | 366 | YES | NO | UN | UN | >9 | 6-9 | NO | 3-6 | >9 | NO | 6-9 | 6-9 |
| 26 | 44 | F | A + | 150 | 150 | NO | YES | 38 | 99 | 3-6 | 3-6 | <3 | 6-9 | 6-9 | NO | 6-9 | 6-9 |
| 27 | UN | F | O + | 179 | 179 | NO | NO | 37.8 | 96 | <3 | NO | NO | 6-9 | 6-9 | NO | 6-9 | 3-6 |
| 28 | 48 | M | A + | 357 | 357 | YES | YES | 39 | 93 | NO | NO | NO | NO | NO | NO | 3-6 | NO |
| 29 | 33 | F | A + | 334 | 334 | YES | NO | 39.1 | 92 | 3-6 | 3-6 | NO | >9 | >9 | NO | NO | >9 |
| 30 | 40 | F | O + | 360 | 360 | YES | NO | 37.7 | 93 | <3 | <3 | NO | >9 | >9 | YES | >9 | >9 |

M: male; F: female; UN: Unknown.

**Supplemental Table 1** (continuation).

| **Patient's ID** | **Signs and symptoms during acute COVID-19** | | | | | | | | | | | | |
| --- | --- | --- | --- | --- | --- | --- | --- | --- | --- | --- | --- | --- | --- |
|  | **Asthenia**  **(months)** | **Anosmia**  **(months)** | **Ageusia**  **(months)** | **Dermatological injuries**  **(months)** | **Odynophagia**  **(months)** | **Dyspnea**  **(months)** | **Pneumonia** | **Pleuritic chest pain**  **(months)** | **Conjuntivitis**  **(months)** | **Diarrhea**  **(months)** | **Dysphagia**  **(months)** | **Abdominal pain**  **(months)** | **Pyrosis /Reflux**  **(months)** |
| 1 | >9 | >9 | >9 | >9 | <3 | >9 | YES | >9 | >9 | >9 | NO | >9 | >9 |
| 2 | >9 | NO | NO | NO | NO | >9 | NO | >9 | NO | NO | NO | NO | 3-6M |
| 3 | >9 | <3 | <3 | NO | >9 | >9 | YES | 6-9 | NO | 6-9 | NO | NO | 6-9 |
| 4 | >9 | NO | NO | <3 | >9 | >9 | NO | >9 | 6-9 | >9 | NO | >9 | NO |
| 5 | >9 | NO | NO | > 9 | NO | >9 | NO | >9 | 6-9 | <3 | NO | 3-6 | <3 |
| 6 | >9 | NO | NO | >9 | >9 | >9 | NO | >9 | <3 | 3-6 | <3 | 6-9 | NO |
| 7 | >9 | <3 | <3 | <3 | >9 | NO | NO | >9 | NO | 6-9 | >9 | >9 | >9 |
| 8 | 6-9 | NO | 6-9 | 3-6 | NO | 6-9 | NO | NO | NO | 6-9 | NO | NO | 6-9 |
| 9 | >9 | <3 | <3 | NO | <3 | >9 | NO | NO | NO | <3 | NO | <3 | >9 |
| 10 | >9 | NO | NO | NO | <3 | >9 | NO | NO | NO | NO | NO | NO | NO |
| 11 | >9 | 6-9 | 6-9 | >9 | >9 | NO | NO | >9 | >9 | >9 | >9 | >9 | >9 |
| 12 | 3-6 | <3 | <3 | <3 | >9 | >9 | NO | >9 | NO | 6-9 | 6-9 | 6-9 | >9 |
| 13 | NO | NO | NO | >9 | >9 | >9 | NO | NO | >9 | NO | NO | NO | >9 |
| 14 | >9 | 3-6 | 3-6 | 3-6 | 6-9 | 3-6 | NO | NO | NO | 6-9 | 3-6 | 3-6 | >9 |
| 15 | >9 | >9 | >9 | NO | >9 | 6-9 | NO | NO | NO | 6-9 | NO | 3-6 | NO |
| 16 | >9 | NO | NO | NO | NO | >9 | NO | >9 | NO | <3 | 3-6 | NO | >9 |
| 17 | >9 | 3-6 | 3-6 | 6-9 | >9 | >9 | NO | >9 | NO | <3 | 3-6 | NO | 6-9 |
| 18 | >9 | <3 | <3 | <3 | <3 | <3 | NO | >9 | <3 | NO | <3 | 3-6 | NO |
| 19 | >9 | NO | NO | NO | NO | NO | NO | >9 | NO | 3-6 | NO | NO | 3-6 |
| 20 | 3-6 | NO | NO | 3-6 | 3-6 | 3-6 | NO | 3-6 | NO | 3-6 | 3-6 | <3 | NO |
| 21 | >9 | >9 | >9 | NO | >9 | >9 | NO | >9 | NO | >9 | >9 | >9 | >9 |
| 22 | >9 | NO | NO | NO | <3 | >9 | NO | >9 | <3 | <3 | NO | NO | NO |
| 23 | >9 | NO | NO | <3 | >9 | >9 | NO | >9 | NO | NO | NO | NO | 6-9 |
| 24 | >9 | <3 | <3 | NO | <3 | >9 | NO | >9 | NO | <3 | NO | NO | NO |
| 25 | >9 | NO | <3 | >9 | NO | >9 | NO | >9 | >9 | NO | NO | NO | >9 |
| 26 | 6-9 | 6-9 | NO | 6-9 | 6-9 | 6-9 | NO | 6-9 | NO | <3 | 3-6 | NO | 6-9 |
| 27 | >9 | NO | NO | NO | <3 | NO | NO | 6-9 | NO | NO | NO | 6-9 | NO |
| 28 | >9 | NO | NO | 6-9 | NO | >9 | NO | NO | NO | NO | NO | NO | NO |
| 29 | >9 | NO | NO | >9 | NO | >9 | YES | >9 | NO | >9 | NO | NO | >9 |
| 30 | >9 | <3 | <3 | <3 | >9 | >9 | NO | >9 | NO | <3 | <3 | <3 | NO |

**Supplemental Table 1** (continuation).

| **Patient's ID** | **COVID-19 Treatment** | **Comorbidities** | | | | | | | | |
| --- | --- | --- | --- | --- | --- | --- | --- | --- | --- | --- |
|  |  | **Autoimmune disease** | **Diabetes mellitus** | **DM onset after COVID**  **(months)** | **Dyslipidemia** | **Arterial hypertension** | **Asthma COPD** | **Cardiovascular disease** | **Hypothyroidism** | **T3/T4 levels altered after COVID**  **(months)** |
| 1 | LVX; AZM; LMWH; CS; HCQ | NO | NO | NO | NO | NO | NO | NO | NO | NO |
| 2 | CRO; LVX; AZM; HCQ; 25(OH)D | NO | NO | NO | NO | NO | NO | NO | NO | NO |
| 3 | AZM; CS; HCQ; 25(OH)D | RA | NO | >9 | NO | NO | NO | NO | YES | NO |
| 4 | NO | NO | NO | NO | YES | NO | NO | NO | NO | NO |
| 5 | AZM; LMWH; CS; 25(OH)D | NO | NO | NO | NO | NO | NO | NO | NO | NO |
| 6 | AZM; LMWH; CS; CH; 25(OH)D | NO | NO | NO | NO | NO | YES | NO | NO | NO |
| 7 | NO | SS | NO | NO | YES | NO | NO | NO | NO | NO |
| 8 | AMC | NO | NO | NO | NO | NO | NO | NO | NO | NO |
| 9 | AZM; LMWH; HCQ; 25(OH)D | NO | NO | NO | YES | NO | YES | NO | NO | NO |
| 10 | LVX; AZM; CS | NO | NO | NO | NO | NO | YES | NO | NO | NO |
| 11 | AZM; 25(OH)D | MCTD | NO | >9 | NO | NO | NO | NO | YES | NO |
| 12 | AMC; CS | RD | NO | NO | NO | NO | NO | NO | NO | NO |
| 13 | NO | NO | NO | NO | YES | NO | NO | NO | NO | NO |
| 14 | AZM; 25(OH)D | RD | NO | NO | YES | YES | NO | NO | YES | 3-6 |
| 15 | CS | NO | NO | NO | YES | NO | NO | NO | YES | >9 |
| 16 | CS | PsO | NO | NO | NO | NO | NO | NO | NO | NO |
| 17 | AZM; CS; 25(OH)D | NO | NO | NO | NO | NO | NO | NO | NO | NO |
| 18 | LVX; AZM; CS | NO | NO | NO | NO | NO | NO | NO | NO | NO |
| 19 | 25(OH)D | NO | NO | NO | NO | NO | NO | NO | NO | NO |
| 20 | CS | NO | NO | NO | NO | NO | NO | NO | NO | NO |
| 21 | AMC; CRO; AZM; CS; 25(OH)D | NO | NO | NO | NO | NO | NO | NO | NO | NO |
| 22 | NO | NO | NO | NO | NO | NO | NO | NO | YES | 6-9 |
| 23 | AZM; CS; 25(OH)D | NO | NO | NO | YES | NO | NO | NO | NO | <3 |
| 24 | AZM; LMWH; HCQ | NO | NO | NO | NO | NO | NO | NO | NO | NO |
| 25 | LVX; CS | PsO | NO | >9 | YES | YES | YES | NO | YES | NO |
| 26 | AZM | NO | NO | NO | NO | NO | YES | NO | NO | NO |
| 27 | NO | HasTh | NO | NO | NO | NO | NO | YES | YES | 6-9 |
| 28 | CRO; LVX; AZM; HCQ; LPV/r; 25(OH)D | NO | NO | NO | NO | NO | NO | NO | NO | NO |
| 29 | AZM; CS; HCQ; LPV/r | PsO | NO | NO | NO | NO | NO | NO | NO | NO |
| 30 | AMC; LMWH; HCQ | NO | NO | NO | YES | YES | NO | NO | NO | NO |

25(OH)D, vitamin D; AMC, Amoxicillin/Clavulanate; AZM, Azithromycin; COPD, Chronic Obstructive Pulmonary Disease; CRO, Ceftriaxone; CS, Corticosteroids; DM, Diabetes mellitus; HasThy, Hashimoto's Thyroiditis; HCQ, Hydroxychloroquine; LPV/r, Lopinavir/Ritonavir; LMWH, Low Molecular Weight Heparin; LVX, Levofloxacin; MCTD, Mixed Connective Tissue Disease; PsO, Psoriasis; SS, Sjögren's Syndrome; RA, Rheumatoid Arthritis; RD, Raynaud's Disease: T3, Triiodothyronine; T4, Thyroxine.

**Supplemental Table 1** (continuation).

| **Patient's ID** |  | **Long-COVID signs and symptoms** | | | | | | | | | | | | |
| --- | --- | --- | --- | --- | --- | --- | --- | --- | --- | --- | --- | --- | --- | --- |
|  | **Bradypsychia**  **(months)** | **Cacosmia**  **(months)** | [**Paresthesia**](https://en.wikipedia.org/wiki/Paresthesia)  **(months)** | **Xerostomia**  **(months)** | **Tinnitus**  **(months)** | **Dysphonia/ Aphonia**  **(months)** | **Earache**  **(months)** | **Hearing loss**  **(months)** | **Diplopia**  **(months)** | **Eyeache**  **(months)** | **Headache**  **(months)** | **Neck/back- ache** | **Poor concentration (months)** | **Memory faillure**  **(months)** |
| 1 | >9 | >9 | >9 | >9 | NO | >9 | >9 | NO | NO | >9 | >9 | >9 | >9 | >9 |
| 2 | >9 | NO | >9 | NO | NO | NO | NO | NO | NO | >9 | >9 | NO | >9 | >9 |
| 3 | NO | NO | >9 | NO | NO | NO | 6-9 | NO | NO | >9 | NO | NO | NO | NO |
| 4 | 3-6 | NO | 6-9 | >9 | NO | >9 | NO | NO | 3-6 | NO | 3-6 | >9 | >9 | >9 |
| 5 | NO | NO | >9 | NO | NO | NO | <3 | NO | NO | NO | 6-9 | >9 | NO | NO |
| 6 | >9 | 6-9 | >9 | NO | >9 | <3 | >9 | NO | NO | 3-6 | >9 | >9 | >9 | >9 |
| 7 | >9 | NO | 6-9 | >9 | NO | >9 | <3 | NO | NO | 6-9 | >9 | >9 | 6-9 | >9 |
| 8 | 6-9 | 6-9 | 6-9 | 6-9 | 6-9 | NO | NO | 6-9 | NO | NO | 6-9 | NO | 6-9 | 6-9 |
| 9 | >9 | NO | NO | NO | >9 | NO | NO | NO | NO | >9 | >9 | >9 | >9 | >9 |
| 10 | >9 | NO | NO | >9 | NO | 3-6 | NO | NO | NO | NO | NO | >9 | >9 | >9 |
| 11 | >9 | >9 | >9 | >9 | >9 | >9 | >9 | >9 | >9 | >9 | >9 | >9 | >9 | >9 |
| 12 | >9 | >9 | >9 | >9 | NO | >9 | NO | NO | NO | 6-9 | 3-6 | >9 | 6-9 | >9 |
| 13 |  | NO | NO | NO | NO | >9 | NO | NO | NO | >9 | >9 | >9 | >9 | >9 |
| 14 | 6-9 | 3-6 | 6-9 | >9 | 6-9 | 6-9 | 3-6 | >9 | NO | 6-9 | >9 | >9 | >9 | >9 |
| 15 | >9 | <3 | <3 | NO | >9 | NO | NO | NO | NO | NO | >9 | >9 | >9 | >9 |
| 16 | >9 | NO | >9 | NO | >9 | NO | 6-9 | NO | NO | NO | >9 | >9 | >9 | >9 |
| 17 | >9 | NO | >9 | >9 | NO | >9 | NO | NO | NO | >9 | >9 | >9 | >9 | >9 |
| 18 | 6-9 | <3 | <3 | NO | 3-6 | <3 | <3 | NO | NO | 6-9 | >9 | 3-6 | 6-9 | 6-9 |
| 19 | NO | NO | NO | NO | NO | NO | NO | NO | NO | NO | NO | NO | NO | NO |
| 20 | 3-6 | <3 | <3 | NO | NO | NO | NO | 3-6 | NO | NO | 3-6 | NO | 3-6 | 3-6 |
| 21 | NO | 6-9 | >9 | >9 | >9 | >9 | >9 | NO | NO | NO | >9 | >9 | >9 | >9 |
| 22 | 3-6 | NO | NO | NO | NO | NO | NO | <3 | NO | 3-6 | 3-6 | NO | 3-6 | 3-6 |
| 23 | 6-9 | 6-9 | >9 | >9 | NO | NO | NO | NO | NO | >9 | >9 | NO | >9 | 6-9 |
| 24 | >9 | NO | 6-9 | 6-9 | >9 | >9 | 6-9 | 6-9 | NO | >9 | >9 | 6-9 | >9 | >9 |
| 25 | >9 | NO | NO | >9 | NO | 6-9 | NO | NO | NO | NO | >9 | >9 | >9 | >9 |
| 26 | 6-9 | NO | 3-6 | 6-9 | 6-9 | 6-9 | NO | NO | NO | 6-9 | 6-9 | 6-9 | 6-9 | 6-9 |
| 27 | >9 | NO | NO | <3 | NO | >9 | NO | NO | NO | NO | 3-6 | >9 | >9 | >9 |
| 28 | NO | NO | NO | NO | NO | NO | NO | NO | NO | NO | NO | >9 | 3-6 | >9 |
| 29 | >9 | NO | >9 | NO | NO | NO | NO | NO | NO | >9 | >9 | >9 | >9 | >9 |
| 30 | >9 | NO | >9 | NO | >9 | >9 | 3-6 | NO | NO | NO | >9 | >9 | >9 | >9 |

**Supplemental Table 1** (continuation).

| **Patient's ID** | **Long-COVID signs and symptoms** | | | | | | | | | | | **Current treatments at the time of sampling** |
| --- | --- | --- | --- | --- | --- | --- | --- | --- | --- | --- | --- | --- |
|  | **Depression** | **Anxiety** | **Insomnia** | **Coagulopathy** | **Petechiae** | **Palpitations** | **Myocarditis/ pericarditis** | **Arrhythmia** | **Urine infection** | **Nail changes** | **Alopecia** |  |
| 1 | NO | NO | >9 | NO | NO | >9 | NO | >9 | NO | NO | NO | Etonogestrel-ethinyl estradiol, buprenorphine, cetirizine, enoxaparin, bisoprolol, ivabradine, cholecalciferol, lorazepam, tramadol |
| 2 | NO | NO | NO | NO | 3-6 | >9 | NO | NO | NO | NO | 3-6 | NO |
| 3 | NO | NO | >9 | NO | NO | NO | NO | >9 | NO | NO | 3-6 | Methotrexate, sulfasalazine, levothyroxine, folic acid |
| 4 | 6-9 | 3-6 | 3-6 | NO | NO | 6-9 | NO | NO | NO | NO | 6-9 | Rosuvastatin |
| 5 | >9 | 3-6 | NO | > 9 | NO | NO | <3 | NO | NO | NO | > 9 | NO |
| 6 | >9 | >9 | >9 | NO | 3-6 | <3 | 3-6 | NO | NO | >9 | 6-9 | NO |
| 7 | >9 | >9 | <3 | NO | >9 | 3-6 | NO | NO | NO | 6-9 | 6-9 | Esomeprazole |
| 8 | 6-9 | NO | 6-9 | NO | NO | 6-9 | NO | NO | <3 | 6-9 | NO | Paracetamol, tramadol, ibuprofen, naproxen |
| 9 | >9 | >9 | >9 | <3 | NO | >9 | NO | NO | NO | NO | NO | Salbutamol |
| 10 | 6-9 | NO | NO | NO | NO | >9 | NO | NO | NO | NO | 6-9 | Budesonide/formoterol, ipratropium bromide, pantoprazole, bromazepam |
| 11 | >9 | >9 | >9 | NO | >9 | >9 | NO | >9 | NO | NO | >9 | Azathioprine, chloroquine, acetylsalicylic acid, escitalopram, cholecalciferol |
| 12 | >9 | NO | >9 | <3 | NO | >9 | NO | NO | NO | NO | NO | NO |
| 13 | NO | <3 | 3-6 | <3 | 3-6 | >9 | NO | NO | NO | NO | >9 | NO |
| 14 | >9 | >9 | 6-9 | NO | 3-6 | 3-6 | NO | NO | NO | 3-6 | 3-6 | Levothyroxine, folic acid, amitriptyline, deflazacort, hydroxychloroquine, omeprazole, cholecalciferol |
| 15 | NO | NO | >9 | NO | >9 | >9 | NO | NO | >9 | NO | >9 | Levothyroxine, duloxetine |
| 16 | >9 | >9 | NO | NO | NO | >9 | NO | NO | NO | NO | NO | Duloxetine, omeprazol, amitriptyline, zolpidem |
| 17 | >9 | >9 | >9 | NO | NO | >9 | NO | NO | NO | 6-9 | 6-9 | Desogestrel/ethinyl estradiol |
| 18 | NO | NO | 3-6 | 3-6 | NO | >9 | NO | >9 | <3 | NO | 6-9 | NO |
| 19 | NO | NO | NO | NO | NO | NO | NO | 3-6 | NO | NO | NO | NO |
| 20 | 3-6 | NO | <3 | NO | NO | 3-6 | NO | NO | NO | NO | 3-6 | NO |
| 21 | >9 | >9 | NO | NO | NO | >9 | NO | >9 | 6-9 | NO | NO | Dexketoprofen, acetaminophen, omeprazole, cholecalciferol |
| 22 | 6-9 | 6-9 | 3-6 | NO | NO | >9 | NO | NO | NO | NO | NO | levothyroxine |
| 23 | NO | NO | >9 | NO | <3 | >9 | NO | >9 | NO | <3 | <3 | Drospirenone/ethinyl estradiol, cholecalciferol, acetaminophen, ibuprofen |
| 24 | NO | NO | >9 | NO | <3 | >9 | NO | NO | NO | NO | NO | Flunarizine, pregabalin, zolpidem, mirtazapine |
| 25 | 3-6 | NO | <3 | NO | NO | <3 | NO | <3 | NO | NO | 6-9 | Levothyroxine, acetylsalicylic acid, cholecalciferol, atorvastatin, amiloride/hydrochlorothiazide |
| 26 | 6-9 | 6-9 | 3-6 | NO | 6-9 | 6-9 | NO | NO | NO | 6-9 | NO | Fluticasone/formoterol, tiotropium bromide, sertraline, paracetamol |
| 27 | 6-9 | 6-9 | 6-9 | NO | NO | 6-9 | 6-9 | 6-9 | 6-9 | NO | >9 | Levothyroxine, cholecalciferol, ferrous sulfate, vitamin B12 |
| 28 | >9 | >9 | 3-6 | NO | NO | NO | NO | NO | NO | NO | NO | NO |
| 29 | NO | NO | NO | NO | 6-9 | NO | NO | NO | NO | NO | 3-6 | Acetylsalicylic acid, folic acid, vitamins B1-B6-B12 |
| 30 | NO | NO | >9 | NO | NO | >9 | NO | NO | NO | NO | 3-6 | Diltiazem, enalapril, atorvastatin, lorazepam |
