# Supplementary material for: Persistent Overactive Cytotoxic Immune Response in a Spanish Cohort of Individuals With Long-COVID: Identification of Diagnostic Biomarkers
Source: Front Immunol. 2022 Mar 25;13:848886. doi: 10.3389/fimmu.2022.848886 (PMC8990790; doi:10.3389/fimmu.2022.848886)
Supplement: Supplementary file 6 [file Table_2.docx]

**Supplemental Table 2.** Clinical characteristics of patients completely recovered from mild COVID-19 that were recruited at the Primary Healthcare Center Doctor Pedro Laín Entralgo (Madrid, Spain).

| **Patient's ID** | **Age (years)** | **Gender**  **(M/F)** | **Blood Group**  **(ABO, Rh±)** | **Days from clinical onset to sample** | **Days with COVID-19 symptoms** | **SARS-CoV-2 qRT-PCR** | **SARS-CoV-2 IgGs** | **Signs and symptoms during acute COVID-19** | | | | | | | | | |
| --- | --- | --- | --- | --- | --- | --- | --- | --- | --- | --- | --- | --- | --- | --- | --- | --- | --- |
|  |  |  |  |  |  |  |  | **Fever**  **(ºC)** | **Cough and expectoration** | **Hemoptysis** | **Odynophagia** | **Dyspnea** | **Pneumonia** | **Pleuritic chest pain** | **Conjuntivitis** | **Diarrhea** | **Vomiting** |
| 1 | 64 | M | O- | 75 | 0 | YES | NO | UN | YES/YES | YES | YES | YES | YES | NO | NO | NO | NO |
| 2 | 57 | F | A+ | 85 | UN | YES | NO | 38.6 | YES/NO | NO | YES | YES | YES | NO | NO | YES | NO |
| 3 | 58 | F | A+ | 73 | 21 | YES | NO | 36.8 | NO/NO | NO | YES | NO | NO | NO | NO | NO | NO |
| 4 | 50 | F | A- | 81 | 38 | YES | NO | 38 | NO/NO | NO | YES | NO | NO | NO | NO | NO | NO |
| 5 | 59 | F | B- | 83 | UN | YES | NO | 38.9 | NO/NO | NO | NO | NO | NO | YES | NO | NO | NO |
| 6 | 53 | F | A+ | 87 | 38 | YES | NO | NO | YES/YES | NO | NO | NO | NO | NO | NO | NO | NO |
| 7 | 41 | F | A+ | 83 | 18 | YES | NO | 37.5 | YES/NO | NO | YES | NO | NO | NO | NO | NO | NO |
| 8 | 29 | M | O+ | 87 | 7 | YES | NO | 37.4 | NO/NO | NO | YES | NO | NO | YES | NO | NO | NO |
| 9 | UN | M | A+ | UN | 0 | NO | YES | UN | NO/NO | NO | NO | NO | NO | NO | NO | NO | NO |
| 10 | 26 | F | A+ | 87 | 3 | YES | NO | 38.5 | YES/YES | NO | YES | YES | NO | YES | NO | NO | NO |
| 11 | 26 | M | UN | 88 | 13 | YES | NO | NO | NO/NO | NO | YES | NO | NO | NO | NO | YES | NO |
| 12 | 28 | M | UN | 79 | 13 | YES | NO | 37.6 | YES/NO | NO | NO | NO | NO | YES | NO | NO | NO |
| 13 | 31 | M | AB+ | 90 | 26 | YES | YES | NO | NO/NO | NO | YES | YES | YES | NO | NO | YES | NO |
| 14 | 47 | F | B+ | 99 | 49 | NO | YES | 37.5 | YES/NO | NO | YES | YES | NO | YES | NO | YES | NO |
| 15 | 62 | M | O- | 82 | UN | YES | NO | 37.8 | YES/NO | NO | NO | YES | NO | NO | NO | NO | NO |
| 16 | 57 | F | A+ | 66 | 16 | YES | NO | 37 | NO/NO | NO | NO | NO | NO | NO | NO | YES | YES |
| 17 | 29 | F | AB- | 83 | 9 | YES | NO | 36.5 | YES/NO | NO | YES | YES | NO | NO | NO | NO | NO |
| 18 | 45 | M | UN | 82 | 10 | YES | NO | 38.5 | YES/NO | NO | NO | NO | NO | NO | NO | NO | NO |
| 19 | 27 | M | O- | 85 | 4 | YES | NO | 37.5 | NO/NO | NO | NO | NO | NO | NO | NO | YES | NO |
| 20 | 26 | F | A+ | 74 | 6 | YES | NO | 37.1 | NO/NO | NO | NO | NO | NO | NO | NO | NO | NO |

M: male; F: female; UN: Unknown.

**Supplemental Table 1** (continuation).

| **Patient's ID** | **Signs and symptoms during acute COVID-19** | | | | | | | | | **COVID-19 Treatment** | **Comorbidities** | | | | | | | | |
| --- | --- | --- | --- | --- | --- | --- | --- | --- | --- | --- | --- | --- | --- | --- | --- | --- | --- | --- | --- |
|  | **Malaise** | **Lethargy** | **Migraine** | **Arthalgia** | **Myalgia** | **Asthenia** | **Anosmia** | **Ageusia** | **Dermatological injuries** |  | **DM** | **DL** | **AHT** | **Asthma or COPD** | **CVD** | **VTE** | **HT** | **Auto**  **immune disease** | **Current treatment** |
| 1 | YES | NO | NO | YES | YES | YES | NO | NO | NO | HCQ, AZM, AMC | YES | YES | NO | NO | NO | NO | NO | NO | Metformin, tamsulosin, atorvastatin |
| 2 | YES | NO | NO | YES | YES | YES | YES | YES | YES | HCQ, AZM, AMC | NO | NO | NO | NO | NO | NO | NO | NO | Eletriptan, diazepam |
| 3 | YES | NO | NO | YES | YES | YES | NO | NO | NO | AZM | NO | NO | NO | YES | NO | NO | NO | NO | Vortioxetine |
| 4 | NO | NO | NO | NO | YES | YES | NO | NO | NO | NO | NO | NO | NO | NO | NO | NO | NO | NO | No |
| 5 | YES | NO | NO | YES | YES | YES | YES | YES | NO | NO | Un | YES | NO | NO | NO | NO | NO | NO | No |
| 6 | YES | NO | NO | NO | NO | YES | NO | NO | NO | NO | NO | NO | NO | NO | NO | NO | NO | NO | No |
| 7 | YES | NO | YES | YES | YES | YES | YES | YES | NO | NO | NO | NO | NO | NO | NO | NO | NO | NO | No |
| 8 | YES | NO | NO | NO | NO | YES | YES | YES | NO | NO | NO | NO | NO | NO | NO | NO | YES | NO | No |
| 9 | NO | NO | NO | NO | NO | NO | NO | NO | NO | NO | NO | NO | NO | NO | NO | NO | NO | NO | No |
| 10 | YES | NO | NO | YES | YES | YES | NO | NO | NO | NO | NO | NO | NO | YES | NO | NO | YES | NO | No |
| 11 | YES | NO | NO | YES | YES | YES | YES | YES | NO | NO | NO | NO | NO | NO | NO | NO | NO | NO | No |
| 12 | YES | NO | NO | NO | YES | YES | NO | NO | NO | NO | NO | NO | NO | NO | NO | NO | NO | NO | No |
| 13 | YES | NO | NO | YES | NO | YES | YES | YES | NO | NO | NO | NO | NO | NO | NO | NO | NO | NO | No |
| 14 | YES | YES | YES | YES | YES | YES | YES | YES | NO | HCQ, AZM, LMWH | YES | NO | NO | NO | YES | NO | NO | SS, APS | Acetylsalicylic acid, HCQ, enalapril, belimumab, escitalopram, prednisone |
| 15 | YES | NO | NO | NO | YES | YES | NO | NO | NO | HCQ | NO | NO | NO | NO | NO | NO | NO | NO | No |
| 16 | YES | Un | YES | YES | YES | YES | YES | YES | NO | NO | NO | YES | YES | NO | YES | NO | NO | NO | Fluoxetine |
| 17 | YES | NO | NO | NO | YES | YES | NO | NO | YES | NO | NO | NO | NO | NO | NO | NO | NO | NO | No |
| 18 | YES | NO | YES | NO | YES | YES | YES | YES | NO | NO | NO | YES | NO | NO | NO | NO | NO | NO | Simvastatin |
| 19 | YES | NO | NO | YES | YES | YES | NO | NO | NO | NO | NO | NO | NO | YES | NO | NO | NO | NO | No |
| 20 | YES | NO | NO | NO | NO | NO | YES | YES | NO | NO | NO | NO | NO | NO | NO | NO | NO | NO | NO |

AHT, Arterial Hypertension; AMC, Amoxicillin/Clavulanate APS, Antiphospholipid Syndrome; AZM, Azithromycin; COPD, Chronic Obstructive Pulmonary Disease CVD, Cardiovascular Diseases; DL, Dyslipidemia; DM, Diabetes Mellitus, HCQ, Hydroxychloroquine; HT, Hypothyroidism; LMWH, Low Molecular Weight Heparin; SS, Sjögren's Syndrome; VTE, Venous Thromboembolism.
